# Validation of an AAV gene therapy designed to complement the loss of TDP‐43 splicing repression for ALS‐FTD

**DOI:** 10.1002/alz.087816

**Published:** 2025-01-03

**Authors:** Aswathy Peethambaran Mallika, Philip C Wong

**Affiliations:** ^1^ Johns Hopkins University School of Medicine, Baltimore, MD USA

## Abstract

**Background:**

Emerging evidence support the notion that loss of splicing repression by TDP‐43, an RNA binding protein that was first implicated in ALS‐FTD, underlies their pathogenesis. Previously, we showed that delivery of an AAV9 vector at early postnatal day expressing a fusion protein, termed CTR comprised of the N‐terminal region of TDP‐43 and an unrelated splicing repressor termed RAVER1 complemented the loss of TDP‐43 in mice lacking TDP‐43 in spinal motor neurons (*ChAT‐IRES‐Cre;tardbp^F/F^* mice). To translate this potential therapeutic strategy to the clinic, it will be important to demonstrate benefit of such AAV delivery of CTR to motor neurons in adult mice. Here we validate a therapeutic approach using a recently developed AAV.PHP.eB vector that enables efficient transduction in central neurons, including motor neurons by an intra‐venous (IV) administrative route.

**Method:**

Intra‐venous injection of AAV.PHP.eB.RAV1‐3’UTR (*ChAT‐IRES‐Cre;Tardbp^F/F^*, n = 12 and *ChAT‐IRES‐Cre;Tardbp^F/+^*, n = 8) and AAV.PHP.eB.GFP (*ChAT‐IRES‐Cre;Tardbp^F/F^*, n = 10 and *ChAT‐IRES‐Cre;Tardbp^F/+^*, n = 8) was carried out in 6 weeks old littermate animals. Histology and immunohistochemistry to show the infectivity, RT‐PCR and RNA in situ hybridization to show the rescue of splicing repression and the grip strength analysis to measure the motor strength were performed.

**Result:**

We show that IV delivery of AAV.PHP.eB.RAV1‐3’UTR to adult *ChAT‐IRES‐Cre;Tardbp^F/F^* mice efficiently transduced spinal motor neurons, repressed cryptic exon inclusion and attenuated muscle weakness.

**Conclusion:**

These data support validation of our AAV gene therapeutic strategy to attenuate motor neuron disease, including ALS and potentially extended to other human disorders harboring TDP‐43 pathology. Other relevant results, including rescue of motor neurons and extension of life span will be presented.

